# The quest for environmental justice in China: citizen participation and the rural–urban network against Panguanying’s waste incinerator

**DOI:** 10.1007/s11625-018-0545-6

**Published:** 2018-03-16

**Authors:** Thomas Johnson, Anna Lora-Wainwright, Jixia Lu

**Affiliations:** 1 Department of Politics, University of Sheffield, Elmfield Building, Northumberland Road, Western Bank, Sheffield, S10 2TU UK; 2 School of Geography and the Environment, Oxford University Centre for the Environment, University of Oxford, South Parks Road, Oxford, OX1 3QY UK; 3 College of Humanities and Development Studies, China Agricultural University, 17 Qinghua E Road, Haidian Qu, Beijing Shi, 100083 People’s Republic of China

**Keywords:** Environmental justice, Sustainability, Environmental networks, China, Waste incineration, Protest

## Abstract

Environmental distribution conflicts (EDCs) related to the construction and operation of waste incinerators have become commonplace in China. This article presents a detailed case study of citizen opposition to an incinerator in the village of Panguanying, Hebei Province. Drawing on in-depth fieldwork, we show how this case was notable, because it transcended the local arena to raise bigger questions about environmental justice, particularly in relation to public participation in siting decisions, after villagers exposed fraudulent public consultation in the environmental impact assessment. An informal network between villagers and urban environmental activists formed, enabling the Panguanying case to exert influence far beyond the village locality. This network was critical in creating wider public debate about uneven power and substandard public participation in siting disputes, a central feature in many Chinese EDCs. By transcending local specificities and exposing broader, systemic inadequacies, this case became instrumental in supporting “strong sustainability”.

## Original research article

Environmental distribution conflicts (EDCs), defined as “mobilizations by local communities against particular economic activities whereby environmental impacts are a key element of their grievances” (Temper et al. 2015: 261–2), have become widespread in China. Whilst they are often centred on community efforts to uphold social justice and protect their local environments, EDCs can also be important by contributing to broader sustainability transitions (Scheidel et al. 2017; Temper et al. 2018; Herrero and Vilella 2017; Camisani 2018). They are driven by changes in social metabolism, namely “the manner in which human societies organize their growing exchanges of energy and materials with the environment” (Martinez-Alier et al. 2010: 153). Although such changes benefit certain groups, others suffer from falling livelihoods, environmental degradation, and worsening public health (Martinez-Alier et al. 2010). Recent transformations in China’s industrial, economic, and social structures have spawned a wide range of EDCs, from large-scale urban protests over facilities such as chemical plants and waste incinerators, to protracted struggles over industrial pollution in rural areas (Lora-Wainwright et al. 2012, 2017; Steinhardt and Wu 2016).

In China, EDCs centred on the construction and operation of waste incinerators have become particularly commonplace. Since 2004 China has been the world’s biggest waste generator, and by 2030 is predicted to generate double the amount of municipal solid waste produced in the United States (Hoornweg and Bhada-Tata 2012). An impending “garbage crisis” (*laji weiji*) has resulted in major restructuring of solid waste disposal. This includes a surge in construction of “waste-to-energy” (WTE) incinerators, some of which can burn thousands of tonnes of waste per day (Johnson 2017).^1^ The Chinese state’s 12th 5-Year Plan targeted a tripling of the number of incinerators, from 103 in 2010 to over 300 by the Plan’s end in 2015 (Johnson 2013: 357). As of February 2016, 231 incinerators were operational in China (Wuhu Ecology Center and Friends of Nature 2016).

WTE incineration is a lucrative sector in China. It features a mixture of state-owned enterprises, Chinese private companies, and multinational corporations and benefits from preferential policies and tax breaks (Johnson 2017). Yet, there has been substantial opposition from communities that bear the brunt of incinerators detrimental to quality of life including, in some cases, public health (Balkan 2012). Through our on-going work on the EJ Atlas project, we identified 54 anti-incinerator EDCs in China. Most of these were identified through an internet search and from information provided by Chinese environmental NGO activists. At time of writing, 11 cases had been entered into the EJ Atlas database (http://ejatlas.org), a collaborative project whose participants collect and upload case studies of social conflict surrounding environmental issues from around the world. These 11 cases are drawn from Beijing (Asuwei, Liulitun), Wuhan (Guodingshan), Guangdong Province (Luoding, Likeng, Panyu, Boluo, Qingshuihe), Hangzhou (Yuhang), Fujian Province (Qingpuling), and Hebei Province (Panguanying). Contention over incinerators in these cases occurred between 2005 and 2014. In eight of these cases, citizen opposition was aimed at preventing incinerators from being constructed, whilst in three of the cases, citizens fought existing facilities. In seven of these cases, community activists forged links (albeit to varying degrees) with Chinese environmental NGOs, particularly Beijing-based Nature University, discussed in more detail below. In more than half of the cases, communities had been suffering from serious pollution for several years, mostly in the form of landfills that existed long before the incinerators. And, seven of the cases resulted in the incinerators being relocated or indefinitely halted, and might, therefore, be considered “successful” cases for local residents. This high success rate is not representative of incinerator-related EDCs in China, however. As Yongshun Cai (2010) has demonstrated, there is often a positive relationship between media coverage of contention and its likelihood of success, because media reporting can expose local injustices to a wider audience and compel higher levels to intervene to shore up regime legitimacy. Yet, access to the media is not easily available to the majority of communities (Cai 2010). This greatly increases the difficulty of obtaining information on these cases so that they can be added to the EJ Atlas database. It is likely, therefore, that many disputes, particularly unsuccessful cases, remain under the radar. Indeed, studies by Chinese scholars suggest that most cases of citizen contention are unsuccessful, and that this is particularly so in rural areas (e.g., Chen 2014; Zhang 2009).

Anti-incinerator conflicts—just like EDCs more broadly—are usefully analysed through the concept of environmental justice. This concept is premised upon the assumption that “generalised social injustices are manifest in environmental conditions, among other ways” (Schlosberg 2013: 40). Early applications of an environmental justice framework focused on the uneven distribution of toxic pollution to racial and ethnic minority and low-income communities in the United States (see for instance Bullard 1990). Since then, the concept has been applied to a growing range of issues, including climate change and biodiversity conservation, and has encompassed aspects of recognition and procedural justice (Schlosberg 2007; Sze and London 2008; Walker 2009). In addition, environmental justice frameworks have increasingly been applied beyond Western liberal democracies (see, for example, McDonald 2002; Williams and Mawdsley 2006; Carruthers 2008; Özkaynak et al. 2015), although rarely so in the case of China (for exceptions see Lora-Wainwright 2017; Ma 2010; Xie 2011). These studies show that the specific contexts in which environmental justice movements develop strongly impacts their ability to trigger broader sustainability transformations (Martinez-Alier et al. 2016; Schneidel et al. 2018). In the United States, for example, disparate campaigns in the footsteps of the infamous Love Canal case scaled up to become a powerful environmental justice movement (Szasz 1994).

This article contributes to the debate surrounding “why, through whom, how, and when” EDCs result in social justice and environmental sustainability (Scheidel et al. 2017) through a detailed study of a Chinese anti-incinerator campaign centred on the village of Panguanying in Hebei Province. This EDC was notable, because more than any other case in our database, it transcended the local arena to raise bigger questions about environmental justice—particularly in relation to public participation in siting decisions—and challenged the desirability of incineration more generally. Crucial to this, was the (strategic) focus on procedural justice and on legal avenues adopted by the key village campaigners. This was facilitated by high levels of interaction between villagers and members of a Beijing-based network of environmental activists comprising NGO activists, legal professionals, academics and journalists, who visited the village and provided support. We know from interviews that the Panguanying case generated a particularly high level of outsider support, which was partly due to the village’s proximity to the capital and the perceived significance of the case according to urban activists. Networks have been analysed as important organisational structures that can help further environmental justice claims and facilitate EDCs, which play a crucial role in promoting sustainability transitions (Schlosberg 1999; Scheidel et al. 2017). Recently, their importance has been highlighted also in the Chinese context (see below for more details). In the case of Panguanying, we show how networking with actors beyond the village helped open legal channels otherwise beyond most ordinary villagers’ reach, and brought the case to a wider audience.

In line with environmental justice literature, the Panguanying case shows how EDCs do not just arise out of distribution of ecological harms, but also due to the uneven distribution of power and participation. Indeed, lack of effective public participation and transparency are regularly contested in Chinese EDCs, and improving them is a key focus of environmental NGO activity. In the Panguanying case, as we shall see, campaigners uncovered evidence of systematic fraud concerning the public participation element of the environmental impact assessment (EIA). This discovery, and the legal contestation surrounding it, was vital to the campaign’s success. Consequently, Chinese environmental activists viewed Panguanying as an exemplary case in highlighting widespread problems associated with the EIA process for waste incinerators and with limited public participation to a wider (national) audience. By transcending local specificities and shining a light on broader, systemic inadequacies, this case became instrumental in supporting “strong sustainability” (Scheidel et al. 2017).

The next section reviews the social movement literature on environmental networks, showing how this relates to what we know about environmental activism in China. We then examine how and why Chinese urban environmental activists have started to forge links with grassroots communities faced with serious pollution threats. The substantive body of the article presents a close and systematic analysis of the Panguanying case. Finally, in the “Discussion” and “Conclusion” sections, the article builds on this original data to elucidate the relationship between EDCs, environmental justice, and sustainability transitions in China.

## Environmental networks

Environmental networks are manifested in a multitude of different forms, ranging from enduring, formal alliances, to “temporary coalitions” between environmental organisations and “not in my backyard” (NIMBY) campaigners (Saunders 2013: 28). In the Chinese context, Wells-Dang (2012) distinguishes between four network types: formal “coalitions” featuring hierarchical structures and large memberships; forums designed for information sharing between members; informal networks that may combine organisational and individual members; and personal networks that exist between individuals.

Although the diversity found in networks can be a source of strength, it can also create tension that undermines collective action (Heyman 2011). Existing studies of environmental activism have highlighted tension between the environmentalism of the wealthy—sometimes linked to Ronald Inglehart’s post-materialist thesis (Inglehart 1977), whereby people only start to care about the environment when they are not concerned with basic material needs—and the “environmentalism of the poor”, where conflicts are localised and rooted in the inequitable distribution of ecological costs and benefits (Guha and Martinez-Alier 2013). Scholarship has also illustrated potential and related tension between locally focused campaigns often aimed at securing compensation (or “weak sustainability”) and broader social movements concerned with “strong sustainability” and premised on the value of the environment per se (see Martinez-Alier 1998, cited in; Scheidel et al. 2017). Harvey, for instance, viewed the “militant particularism” of poor groups as a barrier to social movement formation (Harvey 1996), and Piller argued that mutual suspicion between NGOs and community activists constrained network formation and scale shift (Piller 1991).

But, as the case of Panguanying will illustrate, diversity in aims and environmental discourses initially embraced is not always an obstacle; indeed, it can be transcended in productive ways. Some studies have shown how broad, cross-cultural coalitions that combine, or “hybridize”, different types of environmental discourse can bridge divides between campaigns (Pirkey 2012). Crucially, such encounters do not simply involve a one-way transfer of resources from privileged to poor (as a resource mobilisation approach might imply). Rather, new dynamics and understandings of issues can form through “friction” (Tsing 2005) produced where diverging groups and interests meet. For example, Gottlieb (2001) showed how tension between different manifestations of environmental concern in the United States stimulated the adoption of new tactics and concerns, resulting in the rejuvenation of an environmental movement viewed as overly centralised, hierarchical, and lacking diversity (see also Schlosberg 1999).

China’s political and social context diverges significantly from countries that have been the subject of most environmental justice scholarship, including the United States. For many years, environmental protection was relegated to secondary importance behind economic development. In the past decade, however, environmental issues have risen up the political agenda and now form a crucial element of officials’ evaluation processes (Kostka 2015). The Ministry of Environmental Protection (and its predecessor, the State Environmental Protection Administration), has found common ground with environmental activists committed to improving environmental governance within China’s one-party system (Johnson 2014). This includes efforts to promote greater public participation and transparency as a means to hold local officials accountable for not following environmental laws and regulations. Despite this, many party-state officials remain ambivalent at best to environmental activists, fearing that they may undermine local economic growth or social stability.

Initially, studies on environmentalism in China also examined quests for weak and strong sustainability as though they existed in separation. One group of studies on the latter (strong sustainability) has focused on “environmentalist” NGOs (Johnson 2010), particularly in relation to the issues of nature conservation (Economy 2004; Sun and Zhao 2008) and anti-hydropower campaigning (Liu 2013; Mertha 2008), and in terms of their relationships with the Party-state (Ho and Edmonds 2008). Meanwhile, another body of work has examined how ordinary citizens respond to localised pollution (Jing 2000; Lora-Wainwright 2017; Stern 2013; Tilt 2010; Van Rooij 2010; Van Rooij et al. 2012), how grassroots communities play a role in conservation efforts (Coggins 2003; Hathaway 2013; Herrold-Menzies 2009), and about how communities in ethnic minority areas engage with environmental issues (Yeh 2009). Together, these two groups of studies have (sometimes only implicitly) shown how activism occurs at different points on a spectrum bookended by “embedded in the state” NGOs on the one hand (Ho and Edmonds 2008), and rural pollution victims, who are often “isolated” from intermediary support, on the other (Van Rooij 2010).

While early studies of Chinese environmental activism often made ENGOs their unit of analysis, recent work has focused more on individual activists and networks. Such studies have examined activists’ participation in transnational and regional networks (Wells-Dang 2012; Hathaway 2013; Wu 2013), and how they forge mutually beneficial connections with elites, particularly those within the Party-state (Ho and Edmonds 2008; Spires 2011). Mertha (2008) showed how China’s highly fragmented policy-making process provided a structural opportunity for the formation of anti-hydropower coalitions involving state and non-state actors (Mertha 2008). Wells-Dang (2012), who also examined anti-hydropower campaigning in China, similarly focused on the role of “urban elites”. The crucial role that elite allies can play in environmental contention in China has also prominently featured in the Chinese literature including in relation to anti-incinerator campaigns (e.g., Guo and Chen 2011), but little research has been done on the dynamics of network formation between elites and community activists. One partial exception is Tan and Ren’s (2017) comparison of an environmental NGO and a community action group both involved in a campaign against construction of an incinerator at Asuwei in Beijing. They highlight the large discrepancy in capabilities and resources between these two groups, which resulted in limited cooperation, and advocate closer links between communities and environmental NGOs in future (Tan and Ren 2017). In contrast, we show how community and NGO activists worked together closely in the Panguanying case.

Whilst existing analyses present a significant step forward in identifying potential crossovers between weak and strong sustainability and highlighting the complex interactions between a range of actors, few studies, in Chinese or English, have examined the dynamics of environmental networks in China. Most studies of environmental activism that aims to prevent construction of potentially polluting projects focus on case studies in urban areas (e.g., Chen 2012; Lang and Xu 2013; Johnson 2013). One exception, a study by Bondes and Johnson (2017) which also examines the Panguanying case, pays attention to networks but does not explain in detail how they formed, or explicitly examine their potential contribution to sustainability transitions.

The Panguanying case requires and enables a different type of analysis from these previous studies, one that combines attention to grassroots sensibilities (in this case, those of the villagers), the networks that they became part of, and the ways in which they intersected and interacted. These encounters did not engender tensions but rather the gradual transcendence of the campaign’s significance from the locality itself to a broader anti-incineration network, as their aims converged. Conversely, our article documents mutual feedback between local campaigners and a wide spectrum of urban activists (see Martinez-Alier et al. 2014). The language, focus and strategies used by village-based campaigners drew from insights they gleaned from other successful urban campaigns, but their success also provided crucial support to the agenda of professional environmentalists and environmental lawyers. The case also furnished lessons about the importance of public participation and procedural justice in the quest for strong sustainability.

Our analysis below is based on in-depth interviews with key members of the urban environmental network that we conducted periodically between 2012 and 2017. We also visited Panguanying on several occasions between 2012 and 2016. Johnson interviewed several key village-based campaigners in 2012 after learning about the case from Zhao Zhangyuan, a retired Chinese Academy of Sciences professor and outspoken critic of incineration who had visited Panguanying previously to offer support to villagers (more details below). In 2013, all three co-authors went to Panguanying to conduct further interviews with key campaigners. We employed a snowballing technique and interviewed several other villagers involved in the campaign. However, our efforts to interview a wider range of villagers including some who may not have been directly involved in the case were undermined when local officials in Panguanying told us to cease our fieldwork there. While, as a Chinese researcher, Lu was able to carry out follow-up research and interview a wider range of stakeholders, particularly through employing the help of students, Johnson and Lora-Wainwright had to abandon plans for subsequent fieldwork and limit themselves to interviews with a small number of key villagers who met with us in a nearby city. Our analysis is also based on more than 40 documents, including petition letters and court decisions, which we obtained from leading village activists.

## Pathways to network formation: the urban perspective

Informal networking, based on personal as opposed to institutional linkages, is increasingly recognized as an important feature of China’s environmental movement (Wells-Dang 2012). Beijing is a key hub in this regard. It is home to an informal environmental network (hereinafter referred to as the “Beijing environmental network”) comprising NGO staff, journalists, lawyers, and academics that has advocated, among other things, greater environmental transparency, public participation, and more sustainable solutions to China’s waste challenge.

Whilst Chinese NGOs were previously believed to shun links with pollution victims (Ho and Edmonds 2008), in recent years, Beijing environmental network members have reached out to local communities opposed to incinerators and other forms of pollution. Steinhardt and Wu (2016) claim that media commercialisation and the spread of the internet and social media, declining risks associated with protest, and improved NGO capacity have all facilitated this growing collaboration between policy advocates and protestors. In addition, we find that this development was partly due to frustration among certain environmental activists concerning NGOs’ lack of community engagement, which was part of their “self-imposed censorship” deemed necessary for organisational survival in China’s one-party-state (Ho and Edmonds 2008). In the words of one NGO leader, “my biggest gripe was that NGOs didn’t dare to engage with real issues” (*NGOs bu gan jieru xianshi*). Another important development was the state’s promotion of governance reforms nominally designed to empower the public to hold polluters to account (Johnson 2010). By focusing on procedural deficiencies including perceived lack of official transparency and public consultation, urban middle class protestors shared similar ground with NGOs, yet the latter remained largely disconnected from protests (Johnson 2010).

In this context, environmental journalist Feng Yongfeng established the NGO Nature University (*Ziran Daxue*), which has pioneered a more “grassroots” approach to environmental activism. An organisation in its own right, Nature University has also become a hub, or “clearing house”, for environmentalists in Beijing, for example, through holding regular seminars on environmental issues. Nature University has begun offering help to communities contesting waste incinerators and other forms of pollution. Once contact with a local community is established, Nature University sends staff members to the site with journalists and other “people who care about the issue”. A key aim of this approach is to attract external publicity, or, in other words, to transcend the local context. This serves two main purposes—pressurizing officials into resolving problems through attracting the attention of higher levels and the wider public, and protecting local activists from retribution at the hands of local officials. Instead of advocating disruptive tactics sometimes associated with NIMBY (not in my backyard) campaigns, Nature University encourages communities to utilize “legal weapons” as fully as possible. For example, in another anti-incinerator case at Hai’an in Jiangsu Province, Nature University helped villager Xie Yong, whose son Xie Yongkang was born with cerebral palsy. Xie Yong blamed this on the incinerator located only a couple of hundred metres from his home. Nature University helped Xie Yong contact the Centre of Legal Assistance for Pollution Victims (CLAPV), which represented him in an unsuccessful lawsuit against the incinerator company. Throughout this process, Nature University also helped Xie and his fellow villagers (unsuccessfully) apply for disclosure of the incinerator’s pollution data to use as legal evidence.^2^ As of May 2013, Nature University had filed up to twenty such requests, and sued the Guangzhou Municipal Government when it refused to disclose information about the city’s Likeng incinerator.

Aside from helping pollution victims, Nature University and other activists involved in the Beijing Environmental Network have a more normative goal. In the early 2000s, China’s Ministry of Environmental Protection (MEP) introduced policies to stimulate public participation and transparency, including in the EIA process (Johnson 2014). In helping local residents assert their “right to information, right to supervision, right to participation” (*zhiqing quan, jiandu quan, canyu quan*), network members hope to promote a more participatory form of environmental governance, which addresses the root causes of problems that cause EDCs in the first place. As one NGO leader put it:

We aim to play the role of mediator and be a communication bridge [between the public and the government]… We hope that, after an ignorant appeal (*bu ming bu bai de shangfang*), we can examine why the appeal was ignorant, how to enable public participation, and how to resolve this problem. We need to return to the situation before the appeal took place, and see whether this project can continue. If it can continue, should there be more compensation? Should the site be changed? Is there a need to improve supervision of the facility? *Only if these issues are properly addressed can these NIMBY cases result in a gradual improvement* (*emphasis added*).

Another strand of Nature University’s work involves enabling pollution activists to share their experiences and to network with similar people from other locations. For example, in 2012, a workshop was held on “NIMBY cases in China” that resulted in an NGO report documenting over 20 such “NIMBY” cases.^3^ The recent popularisation of social media and micro blogging—especially Weibo—has also facilitated communication between grassroots communities and urban activists (Bondes and Johnson 2017). Through this, otherwise “isolated” (Van Rooij 2010: 76), activists become networked into a broader activist community where they can share knowledge and experience. One NGO informant described Weibo as a “small command centre” (*xiaoxing zhihuibu*) that serves as a platform for sharing information and breaking issues out of local confines.

Network formation between urban and rural activists is not just a top–down process. It is contingent upon several local level factors, including grassroots leadership. We examine these factors next through a case study of the Panguanying anti-incinerator campaign.

## The Panguanying anti-incinerator campaign

In 2008, the Qinhuangdao Municipal Government decided to construct a waste incinerator in the village of Panguanying, approximately 35 km away from the city yet only 200 metres from the nearest homes. Villagers were excluded from the decision-making process and denied even the most basic information about the project. Instead they were viewed as potential obstacles to be overcome rather than as participants in the decision-making process. The decision-making process was deliberately structured to guarantee support from village leaders and neutralize potential opposition from local people through denying them a voice. Although local officials advertised a public comment period in line with EIA Law requirements, they did this through placing small notices in the county government, which were not seen (and not meant to be seen) by villagers. Then, in early 2009, with the project having been approved “in principle” by various local government departments, inspection trips to other incinerators operated by Weiming, the private company in charge of constructing and operating the Panguanying incinerator, were arranged. Although the Hebei Provincial Environmental Protection Bureau (EPB) subsequently claimed that these visits had successfully dispelled any fears concerning incineration that residents might have had, participants only comprised a handful of local officials who were likely to approve of the project or at least not oppose it. The only village representative included on these trips was Panguanying Party Secretary Qiao Yanli, who had been accused of corruption and was deeply unpopular amongst a significant portion of villagers.

The Panguanying incinerator only became common knowledge in mid-April 2009 when Qiao led a group of workers to measure the land for the project. He was approached by Pan Zuofu, a member of one of Panguanying’s “teams” (the smallest administrative designation which refers to a sub-village unit), which controlled 14 *mu* (a little less than a hectare) of farmland designated for the incinerator. Pan Zuofu became involved in a heated discussion about compensation levels, which he found thoroughly inadequate. Qiao retored that, with higher levels backing the incinerator project, any resistance would be “futile”.

Pan Zuofu enlisted support from his uncle Pan Qingwen, who belonged to the same farming team and whose interests were, therefore, also under threat, as well as other sympathetic villagers. They appealed at multiple levels via the letters and visits (*xinfang*) system that provides an institutionalised channel through which citizens can lodge complaints against officials, claiming that the land expropriation was illegal and that the decision-making process contained procedural flaws. They claimed that their land was “basic farmland” (*jiben nongtian*), and not, as local officials claimed *yuandi* (literally, “garden land”). This mattered because according to Chinese law expropriation of the former requires central government approval whilst the latter does not. However, local officials rebuffed the men’s complaints, and the Hebei EPB approved the project’s EIA in May 2009, allowing construction to begin. Undeterred, the villagers repeatedly contacted the National Land Resources Ministry, which in September 2009 compelled the local county government to halt the project until proper procedures related to the land issue had been followed. Shortly thereafter those procedures were completed and construction resumed.^4^

### Reframing opposition: from land to health

The incinerator issue was soon reframed from a land dispute involving a handful of affected villagers to a major public health issue. First, a villager formerly employed as an environmental protection officer for a local paper mill warned that burning waste could cause the release of dioxins, a harmful pollutant. Then, on 1 September 2009, China Central Television (CCTV) aired an episode of *Half Hour Economy* (Jingji Ban Xiaoshi) entitled *Dioxins are Tormenting China* (Er’eying Kunrao Zhongguo).^5^ A major international conference on dioxins had recently been held in Beijing, where several recent anti-incinerator protests motivated in part by fears concerning dioxin emissions had occurred (Johnson 2013). The *Half Hour Economy* episode dramatized the link between dioxins and cancer, and interviewed Professor Zhao Zhangyuan. The episode also featured interviews with anti-incinerator campaigners from Liulitun, a Beijing suburb where residents had been opposing a planned incinerator since 2006. Through this, Panguanying villagers discovered that other communities were also struggling against incinerators. Importantly, the realization that the incinerator could threaten public health inspired a third villager, Pan Zhizhong, to join the campaign. He distributed copies of *Dioxins are Tormenting China* and other materials from the internet to local people to raise awareness. This resulted in 1500 villagers (from Panguanying and surrounding villages) signing a petition against the incinerator (Shang 2013). Pan Zhizhong then visited every village within a 5-km radius of the incinerator site and collected handwritten statements from 37 village heads expressing opposition to the project.^6^

The Liulitun case strongly influenced Panguanying villagers. There, campaigners had articulated persuasive, facts-based arguments against the incinerator project (Johnson 2013). Liulitun residents produced a detailed 44-page report [hereinafter referred to as the “Liulitun Report”] outlining their rationale and strategies for opposing the incinerator, and uploaded it to the internet. They also fully utilised legal channels, challenging the project based on errors in the siting process such as factual mistakes and lack of public participation (Johnson 2013). They did this through hiring environmental lawyer Xia Jun, who helped campaigners contest the project’s EIA through an administrative review application. They also peacefully surrounded MEP headquarters in Beijing on World Environment Day 2007 to demand the problem’s resolution. In response, the MEP called for the project’s suspension pending further investigation, and the planned incinerator was later relocated (Johnson 2013).

Reflecting on the *Half Hour Economy* episode, one leading campaign participant said,

Lots of national experts said that it’s best to keep incinerators to a minimum because they are harmful to health… We subsequently saw the [Liulitun report], and downloaded it from the Internet…we saw that Liulitun residents had filed a lawsuit, and there was a lawyer, Xia Jun. We decided to also contact a lawyer, because [in relation to the Liulitun Report] we can’t write this kind of thing, we don’t understand environmental law, so we contacted Xia Jun.

In June 2010, Panguanying campaigners produced their own ten-page report.^7^ It resembled the Liulitun document, including scientific assertions that incinerators pose a health hazard and claims that, by excluding the public, the siting decision contravened laws and regulations. Then, in summer 2010, the Pans travelled to Beijing to meet with Zhao Zhangyuan and Xia Jun. Both men, together with several Nature University activists, subsequently visited Panguanying to provide technical information about the dangers of incineration and advise the Pans about how to contest the incinerator. Lawyer Xia agreed to represent the villagers, and in September 2010 helped them file an administrative review application challenging the Hebei EPB’s decision to approve the incinerator project’s EIA on five grounds:

- The project had not been included in municipal government plans;
- The project contravened national government policies, including protection of groundwater and arable land, and of a nearby scenic zone;
- There was no evidence about how dioxin emissions would be kept within safe standards;
- The EIA did not consider, among other things, incinerator sludge and ash treatment, and monitoring of dioxins;
- The EIA violated the law because public opinion wasn’t solicited and there was insufficient information disclosure.

The MEP accepted the administrative review application but ultimately upheld the EPB’s decision. Villagers continued to file administrative reviews into other aspects of the case, including the land designation issue, yet these efforts were also rebuffed. Then, in 2011, again with Xia’s help, they filed an administrative litigation lawsuit at the Shijiazhuang Qiaoxi District People’s Court against the Hebei EPB’s decision to approve the EIA. The case was accepted and both sides submitted evidence in advance of the court case. During that process, villagers stumbled on a discovery that transformed the case.

### Fraudulent public participation: the smoking gun

The China Meteorological Association (*Qixiang Ju*) (CMA) had conducted the EIA. In evidence submitted to the MEP during the 2010 administrative review process, the provincial EPB claimed results from 100 questionnaires issued to local residents as part of the EIA process indicated “strong public support” (*jun tongyi*) for the project. Residents suspected foul play, but lacked evidence to verify their suspicions. Amazingly, however, this changed when, during the evidence-collecting period for the administrative litigation lawsuit, the plaintiffs obtained the full EIA report from the EPB. According to Xia Jun, the court was known to be relatively strict, and would likely not allow either side to add evidence after proceedings began. He speculated that this explained why the EPB, perhaps unaware of any problems with the EIA, submitted the full version as evidence. After obtaining the EIA, the anti-incinerator campaigners quickly determined that the questionnaire survey had been completely falsified, providing them with an incontrovertible “smoking gun” that proved procedural malpractice. For example, some survey responses had been attributed to villagers who had died or left the village long ago. The 64 “respondents” still living in the village denied participating in the survey. They signed statements indicating that they opposed the incinerator, and that the questionnaires were fake. Upon receiving this news, villagers notified Nature University, which wrote to the MEP and contacted a *Phoenix Weekly* journalist who visited Panguanying and reported on the case (Bondes and Johnson 2017).^8^

Evidence concerning the fake questionnaires proved decisive in stopping construction of the incinerator for a second time. In March 2011, the Hebei EPB halted the project and announced that it would not approve any EIAs in Qinhuangdao until the new incinerator EIA had been passed with proper public consultation. On 27 May, the Hebei EPB officially revoked the EIA, and 2 weeks later the court confirmed that villagers had withdrawn their lawsuit. As of October 2017, construction has not resumed, and villagers employed by Weiming to watch over the half-built incinerator have begun to cultivate vegetables again within its walls.

### The aftermath: negotiating a stalemate

Whilst the halting of the incinerator project was regarded as a major victory for campaigners, the half-built structure still literally and figuratively loomed over the village. Campaigners worried that the EIA would be rectified, allowing the project to resume as planned. Rather than claiming an outright victory then, campaigners’ efforts had resulted in an uneasy stalemate (*jiangju*).

The Hebei EPB’s revoking of the EIA did not, therefore, spell an end to the EDC. In March 2011, villagers challenged the MEP to hold the CMA accountable for the shoddy EIA, and attempted to sue the MEP when it refused to do so. However, the court case was not accepted. Several Beijing-based NGOs were also committed to improving the performance of units conducting EIAs. For example, after discovering similar problems with public consultation for another EIA—also conducted by the CMA—in relation to the Sujiatuo incinerator in suburban Beijing, five NGOs issued an open letter calling for the CMA’s licence for conducting EIAs to be stripped. This call was not heeded. Villagers also challenged Weiming’s application to conduct an initial public offering (IPO). Companies are required to obtain approval from the MEP before launching an IPO to prevent highly polluting companies from being listed; Weiming had requested MEP approval in December 2010. Villagers claimed that the fraudulent EIA rendered Weiming unfit to launch its IPO. However, the MEP announced that, because the IPO application had occurred before the problems with the Panguanying case emerged, Weiming was eligible to proceed with its IPO. These examples highlight the difficulties associated with translating local case-based issues into wider struggles.

Back in Panguanying, in 2012, Pan Zhizhong stood for election as village head after villagers had forced Qiao Yanli to step down. Pan believed that this would enable him to deal a fatal blow to the project. Several environmental activists went to Panguanying to witness the election and offer protection to Pan, who was coming under severe pressure from pro-incinerator forces. However, this election, and a subsequent one, was disrupted by local thug allegedly linked to Township officials, leaving the village without a head for several years.

In late 2012, in a clear signal that they wanted to resume the project following 2 years of inaction, Weiming attempted to negotiate with Pan Zhizhong and Pan Zuofu. The two men agreed to have lunch with Weiming representatives, during which Pan Zhizhong reasserted his intention to stand for village head and oppose the incinerator (Shang 2013). Weiming also invited members of the environmental network—including Mao Da, Chen Liwen, Feng Yongfeng, Zhao Zhangyuan, and Xia Jun—to visit an incinerator in Jiangsu Province, which they accepted. However, scope for cooperation between the two sides was limited. When Weiming asked Mao Da if network members could mediate between it and the villagers, he insisted that the format should be a waste management forum where all issues could be openly debated and covered by the media. However, Weiming refused, and mediation failed, leaving the issue unresolved.

## Discussion

Several factors contributed to the formation of a loose network between village-based activists and a range of anti-incineration activists and experts, which in turn enabled the case to transcend its local significance and become regarded as a ‘classic’ case that activists could learn from. Most basically, local campaigners’ focus on participation and procedural justice allowed their interests to merge with those of professional anti-incineration campaigners. Framing environmental justice claims in the language of procedural justice is a well-practiced strategy for Chinese environmental activists, and belongs to a broader tendency to depoliticise environmental politics (Ho and Edmonds 2008). Indeed, much contention in China relies upon identifying and drawing attention to misimplementation of laws and regulations and taking advantage of cleavages within the state (O’Brien and Li 2006). Official state support for public consultation in relation to siting decisions enabled villagers to challenge the project on procedural grounds, since they had not been adequately consulted. This was also vital in supporting the development of this rural–urban anti-incineration network. Legal reforms promoting public participation are enabling NGOs to outgrow their reliance on personal connections with party-state officials, or, in Ho and Edmonds’ (2008) words, their “embeddedness” and to work more closely with affected communities. Contingent political circumstances also presented a timely opportunity: activists attributed the Shijiazhuang court decision in favour of the villagers to the prior departure of the Provincial EPB Head. This meant that the EIA approval could be suspended without causing him to lose face.

Closely related to this convergence of focus is a convergence of language between village activists and professional environmentalists who collaborated with them. Very much in this vein, village-based campaigners in Panguanying adopted some of the discourses of justice and recognition employed by members of the Beijing anti-incineration network. For example, one villager stated that:

When we sued the government, we didn’t want money, we wanted accountability. Even if they give me one *Yuan*, I don’t mind, as long as the government loses. We can ring an alarm bell in China, that public participation is being incorrectly carried out, so that when other places build something they have to consider public participation, obtain everyone’s agreement. We don’t want money, we’re interested in justice.

Similarly, despite limited opportunities for networking with other communities, leading campaigners in the Panguanying case publicly shunned the “NIMBY” label and acknowledged the wider (strong) sustainability issues concerning waste management. One campaigner stated that he wanted the half-built incinerator to be transformed into an environmental protection museum. And, in August 2017, a Nature University activist involved in the Panguanying case revealed she was discussing the possibility of using the village as a site for a pilot project in waste reduction and sorting with residents. Overall, as the Panguanying case shows, resorting to discourses of sustainability and justice serves as an increasingly desirable strategy for local campaigners to avoid the stigmatised NIMBY label. It also strengthens opportunities for collaboration with urban-based, professional environmentalists including Beijing anti-incineration network members. Conversely, members of the network performed the role of ‘elite allies’ shining a spotlight on the village and exposing official malfeasance.

The charisma and strength of character of the people involved are crucial to the survival of any campaign, let alone its success and the possibility of networking with other campaigners. Although Pan Qingwen was incapacitated by a stroke early in the campaign, Pan Zhizhong and Pan Zuofu resolutely maintained their opposition. This incurred a high personal cost. They were threatened by local thugs and Pan Zuofu’s windows were smashed. Local officials also (unsuccessfully) attempted to persuade the three men to drop their campaign through applying pressure through relational ties, something that Deng and O’Brien (2013) refer to as “relational repression”. For example, one county leader allegedly contacted Pan Zhizhong through a relative, and offered him alternative accommodation outside of the village. Pan Zuofu’s cousin, a civil servant, advised him against continuing his opposition. But, both men refused to listen to these offers/threats. Meanwhile, the Qinhuangdao Government offered to find work for Pan Qingwen’s five daughters, and to pay for treatment for his sick wife—he also refused this offer. When Pan Qingwen’s application for minimum living guarantee was turned down, his daughters claimed that it was because of his involvement in the anti-incinerator campaign.

Opportunities for contact and mutual learning were central to supporting and protecting village-based activists, and in turn to the formation of this rural–urban network. Technology and infrastructure played a key role in this regard. The proximity to Beijing (2 h by high-speed rail), facilitated several face-to-face interactions between villagers and ENGO activists. The media, particularly the CCTV documentary, was also instrumental in supporting the rural–urban network. First and foremost, it made villagers realise that theirs was not an isolated struggle. Second, it sounded alarm bells about the potential health effects of incineration in their vicinity, enabling their opposition to go beyond initial economistic concerns about loss of land and livelihood and to include concerns with health too. The latter provided a crucial shared ground with professional activists. Third, it provided a roadmap for their opposition to the incinerator based on the experience of Liulitun campaign and allowed them to identify and contact potential allies (such as lawyer Xia Jun).

While the formation and persistence of environmental networks requires and fosters some shared ground between the parties involved, it does not necessarily entail that they should share all their aims. Village campaigners were not primarily concerned with the kind of normative governance goals prioritised by Beijing Environmental Network members. As one NGO activist with close ties to the case commented, “[the villagers] just want to pull this project down once and for all”. In addition, apart from focusing on procedural issues, Panguanying villagers also framed their grievances in the language of distribution and recognition. This was evident in their ten-page report, the final section of which was titled “Common People’s Opinions” (*Baixing Yijian*). It drew a sharp distinction between urban areas as producers of waste that were being beautified, and rural areas as dumping grounds populated by irrelevant and disposable people. One passage stated that,

Villagers aren’t a group of fools, we also know to stand up and fight when our survival is threatened. Waste incineration came about because of cities. It cleaned city spaces whilst polluting vulnerable villagers. Almost every aspect [of incinerators sited in rural areas] affects nearby villagers’ livelihoods, sacrificing their health in the short term to alleviate the waste problems that accompany urban development… We also want to survive, we also want environmental protection, and we also want to live with dignity.

Villagers’ examination of official documents concerning the siting decision revealed that Panguanying was chosen, because it was downwind from urban (*chengzhen*) areas and “some distance” (*yiding juli*) from residential areas, which was desirable in limiting “disturbance” (*ganrao*). Yet, according to villagers, almost 30,000 rural residents lived within five kilometres of the incinerator site. This prompted one of the leading campaigners to ask rhetorically “are we not people too?”

In contrast, professional environmentalists seek to use grassroots cases to shape an ambivalent and contested regulatory landscape (Van Rooij et al. 2016). For example, Xia Jun had a vested interest in promoting the resolution of environmental disputes via legal means to expand the role of lawyers in environmental governance.^9^ Recounting a conversation with Peking University law professor Wang Jin, he stated,

we had been discussing law and public participation, we wanted to find some cases, then [the Panguanying] case came along, it was very “classic” (*dianxing*).” I asked him why can’t lawyers help constructors [of projects with potential environmental impact] with [conducting] public participation, which has been very problematic? But we feel it’s difficult, because the MEP and EPBs tend to reject lawyers, they think that the EIA system can handle everything, including legal service. We didn’t have a good example case to show our perspective. But the Panguanying case was one such case, it’s very classic, down to the level of fabrication [of the questionnaires], there was serious illegality.

Similarly, a Nature University staff member repeatedly suggested that Panguanying was a good “case” (*anzi*) from which to learn and for highlighting shortcomings in the EIA process and encourage other communities to also use legal channels to resolve EDCs. One of the main goals of urban activists is to raise societal awareness by enabling cases to enter the public sphere (*jinru shehui de shijiao*). The story of the faked questionnaires was highly newsworthy. Several media outlets, including the *People’s Daily*, reported on the fake questionnaires in an article entitled “EIA Unit Actually Engages in This Kind of Forgery” (*Huanping jigou jing zheyang zaojia*) after environmental activists tipped them off, and Zhao Zhangyuan wrote a blistering criticism of the project’s EIA process.^10^ In addition, Pan Zhizhong and Pan Zuofu have participated in seminars organised by Beijing activists. The first time this happened was in 2010, after Xia Jun introduced the case to other network members. The two men travelled to Beijing and shared their story with activists and other people fighting against pollution. In 2013, shortly after our field visit, the two men participated in a small conference in Beijing on “NIMBY in China”, organised by the NGO Nature University. Their story was written up, along with 20 other cases, and included in the conference proceedings. Participation by local campaigners in such events organised by brokers and urban professional activists is central to the establishment and strengthening of networks.

An important way in which individual, local campaigns become scaled up to broader significance is in their role as precedents. Several precedents were set in the Panguanying case, including the release of the full EIA report and the decision to revoke the EIA approval after the discovery of malpractice in relation to the solicitation of public comments. Given that public participation requirements are frequently overlooked—something that is not helped by weakly worded legislation—Nature University, Xia Jun and other network members hoped that this case could highlight this problem and stimulate broader institutional reforms.

Whilst the network analysed in this article contributed crucially to the relatively “successful” conclusion of this case from the activists’ perspective, it was not the only factor. Panguanying’s proximity to Beijing, just two hours away by train, facilitated the involvement of environmental activists and the media in this case. There was also an element of luck—for example, the EPB’s decision to hand over the entire EIA report came as a surprise to Xia Jun (Bondes and Johnson 2017). And, one NGO activist who had participated in the case told us that waste disposal was not a particularly pressing issue in the region, and that local officials were, therefore, not in a rush to construct an incinerator, something which contributed to the stalemate described above. Finally, the courage and determination of key village activists cannot be underestimated. All of these factors contributed to the outcome in this case.

## Conclusion

Let us return to one of the questions leading this special issue: “why, through whom, how and when” do EDCs result in social justice and environmental sustainability (Scheidel et al. 2017) considering the Panguanying case and the network which formed around it. Rural–urban environmental networks like the one surrounding Panguanying are a new form of movement organising in China. Schlosberg (1999: 142) argued that “networks expand the notion of environmental locality, as they expose the similarities shared by communities in disparate places”. Similarly, in the Panguanying case, their most crucial effect is enabling what began as a place-based EDC to transcend the local level, giving it wider significance and potential to become a precedent for subsequent campaigns. From the view point of local activists, the rural–urban network amplified their voice and helped them challenge unjust decisions. Conversely, this network served the interests of professional activists by creating wider public debate surrounding the misimplementaion of environmental regulations, mainly in relation to public participation in siting disputes, something viewed as an endemic issue in China.

Rural–urban networks like the one which emerged around Panguanying’s incinerator can go far beyond environmental issues in challenging multiple forms of domination (Schlosberg 1999). In the Panguanying case, the battle for recognition was rooted in the wider issue of citizenship and participation. These features of environmental justice are closely linked—as Schlosberg (2007: 25) noted, “if you are not recognized, you do not participate; if you do not participate, you are not recognized”. Decision makers strived to render villagers invisible—they were not informed of the project and were completely excluded from the decision-making process, culminating in the forged questionnaires. By fabricating locals’ approval, the EIA attempted to silence villagers while pretending that they had in fact been given a voice. Conversely, efforts by village campaigners and their allies in Beijing aimed to unmask injustices, both in terms of the lack of recognition of villagers’ views and potential effects on their health and livelihoods.

In discussing the potential for EDCs to contribute to strong sustainability, Scheidel et al. (2017) differentiate between intermodal and intramodal conflicts. The former emerge within an established pattern of resource use between different social groups, whereas the latter defends a particular mode of resource use against industrial society’s attempts to transform it. The Panguanying case straddled these two categories. On the one hand, the village campaigners’ primary goal was to prevent an incinerator being constructed in Panguanying, indicating an intermodal conflict. Yet, in doing so, they became increasingly aware about the dangers of incineration in general, and about shortcomings in the EIA process that robbed them of any agency. This intramodal aspect of the Panguanying case was also influenced by strategic imperatives and by urban activists whose concerns about incineration and environmental governance went far beyond the village itself. Engagement of activists such as the Pans can be both inter and intramodal, depending on the context—the two are not mutually exclusive.

The Panguanying case is an instance in which an EDC supports sustainability. This is not because the incinerator is on hold, but rather because it raises questions about the failings of the EIA process and engages with the question of incineration safety and desirability as a whole. Their proponents claim that waste incinerators represent a clean and efficient way of handling waste, and are preferable to landfill. Yet, studies have cast doubt on this in China due to concerns that incinerators are not managed or regulated to high enough standards (Johnson 2013). One rare study of emissions from 19 Chinese incinerators found considerable variation, with some achieving EU standards and others failing to meet much laxer national standards (Ni et al. 2009). By becoming an exemplary case trumpeted by professional activists and the media, Panguanying highlighted serious problems with the EIA process and showed how the rights and interests of vulnerable populations can be overlooked by planners. And as of late 2017, Nature University activists were in discussion with Panguanying residents about using the village as a pilot site for a rural waste reduction and sorting project. At least three other cases in our sample—Aobei, Liulitun and Panyu—also resulted in community efforts in waste sorting and reduction (Johnson 2013). The case, therefore, supports Scheidel et al. (2017) argument that environmental justice success can support wider efforts to improve sustainability.

Despite Panguanying case’s significance, the prospects for a scaling-up of anti-incinerator activism in China are highly constrained. The professional activist community is small and lacks resources. More importantly, the party-state maintains a strong aversion to linked up activism, particularly between disparate affected communities, that may challenge its monopoly on political power. Hence, whilst there appears to be strong demand among grassroots and activist communities for a strong environmental justice movement based on incinerators and other pollution problems, the prospects for this materialising appear bleak. The vast majority of EDCs will likely remain rooted in local power struggles. Yet, as the early stages of the Panguanying campaign showed, materials related to other communities’ EDC experiences are still readily accessible through the Internet. These discourses, which are rooted in broader concerns about the regulation and governance of environmental risks, will likely inform future EDCs even when campaigners fail to network with outside activists.

The level of networking in the Panguanying case exceeded that witnessed in most of the other anti-incinerator cases documented so far in the EJ Atlas database. One reason why EDCs so often remain localised is that the rural grassroots level often provides an inhospitable environment for activism, even when it is perfectly legal. One urban environmental activist recounted his experience in rural China:

I’m terrified to death of taking a photo [of pollution]. I don’t even dare to call the pollution reporting hotline. It’s like if you make a phone call you are committing a crime. It’s also not possible to tell the police. Sometimes people give me photos and ask me to report the situation, they are scared that the Public Security Bureau will find them if they publicise the photos, they are scared of being arrested.

Because outside attention can result in local officials being disciplined, they have a strong incentive to limit contact between local campaigners and outsiders. The limitations we faced whilst carrying out fieldwork may serve as additional illustration of these dynamics.

Environmental networks face obstacles both against horizontal expansion (forging links between affected communities) and vertical expansion (between professional activists and affected communities) (Bondes and Johnson 2017). Regarding the latter, whilst the MEP introduced regulations to increase the scope of non-state involvement, in the Panguanying case, it repeatedly turned a deaf ear to villagers’ appeals, choosing instead to side with local officials. Even when the CMA had been guilty of serious wrongdoing on more than one occasion, the MEP did not take formal disciplinary action against it. The state’s ambivalence towards non-state actors (Stern 2013)—even when they have legitimate demands—limits the potential for improvements in environmental governance within a one-party system. This is unfortunate, especially given that elite allies such as NGOs, journalists and lawyers help soften the boundaries between state and society and generally view their role as mediators instead of agitators.

As Temper et al. (2018) argue, EDCs can result in the creation of new norms and institutional structures that alter power relations resulting in “transformations to sustainability”. This happens when EDCs coalesce into bigger movements that “question the broader structures causing environmental injustices” and whose approach “is often radical and broad-minded” (Scheidel et al. 2017). In China, any such transformations are most likely to occur when there is strong cooperation between state and non-state actors. Whilst a form of “depoliticised” (Ho and Edmonds 2008) environmental activism is tolerated by the state, this does not include radical movements intent on reconfiguring power structures. For example, the battle to improve public participation in environmental issues is an iterative process between sympathetic state actors and their policies, and non-state activists who demand that those policies are adhered to. Yet, whilst Chinese leaders have thrown their weight behind energy efficiency and pollution reduction policies, making them key aspects of local official evaluation (see Wang 2013; Kostka 2015), the promotion of public participation as a means for enhancing environmental justice lacks high level political support. Although there are several challenges to EDCs scaling-up, the emergence of networks like the one surrounding Panguanying, however contingent, offers some room for hope in the pursuit of strong sustainability.

## Footnotes

1 The city of Shenzhen is currently constructing the world’s biggest waste-to-energy incinerator, which will burn 5,000 tonnes of waste every day (South China Morning Post 2011). The 2008 Trial Measures on Environmental Information Disclosure enable citizens to apply for disclosure of certain types of information.

2 The 2008 Trial Measures on Environmental Information Disclosure enable citizens to apply for disclosure of certain types of information.

3 On file with the authors.

4 One key stakeholder that we interviewed insisted that officials had covertly altered the land designation from basic agricultural land to yuandi in order that these procedures could be completed.

5 The online link to this programme no longer works. It is possible to find an abridged, five-minute version on the Internet (available at http://my.tv.sohu.com/us/5223036/3628761.shtml).

6 After coming under pressure from the Township government, two village heads revoked their complaints (Shang 2013).

7 On file with the authors.

8 See http://news.ifeng.com/fhzk/detail_2011_03/01/4905732_0.shtml.

9 For more information on the mixed motives of environmental lawyers, see Stern 2013 Chap. 6.

10 http://zhaozhangyuan.blog.sohu.com/165521477.html.
